# Oral Cancer Knowledge, Attitude, and Practice of Dentists in the State of Qatar

**DOI:** 10.3390/dj7020043

**Published:** 2019-04-11

**Authors:** Diana H. Jboor, Mohammed S. Al-Darwish, Ula Nur

**Affiliations:** 1 Department of Public Health, College of Health Sciences, QU Health, Qatar University, Doha 2713, Qatar; dj1600061@student.qu.edu.qa; 2 Hamad dental Center, Hamad Medical Corporation, Doha 3050, Qatar; maldarwish@hamad.qa

**Keywords:** oral cancer, knowledge, attitude, practice, dentists, Qatar, continuous professional development courses

## Abstract

Objectives: The aim of this study was to assess existing knowledge on oral cancer (OC), attitude toward OC examination, and clinical practice among dentists practicing in the governmental health sector in the State of Qatar, including the influence of personal characteristics. Materials and Methods: All 271 dentists practicing in Primary Health Care Centers (PHCC) and the Hamad Medical Corporation (HMC) were invited to participate in this cross-sectional study. Of these, 177 completed a self-administered, 48-item questionnaire. Based on the responses, knowledge of the risk factors for and clinical presentation of OC was categorized as high, medium, or low, and then further separated into satisfactory (medium/high) and unsatisfactory (low). Information on attitudes toward OC examination and clinical practice was also taken from the questionnaire. Results: The mean score for knowledge of the clinical presentation of OC was 7.59 (standard deviation [SD] = 2.40) out of 14. The mean score for knowledge of the risk factors for OC was 8.96 (SD = 2.31) out of 16. Dentists with ≤10 years of experience were more likely to have satisfactory knowledge of OC compared to dentists with >15 years of experience. Attending a continuous professional development (CPD) course on OC showed a trend with satisfactory clinical knowledge, although it was not statistically significant. Conclusion: This study identified gaps in dentists’ knowledge of OC; dentists demonstrated unsatisfactory knowledge of the clinical presentation of and risk factors for OC. The findings highlighted the need for educational interventions on OC, which are essential to improving health care outcomes and delivery of care.

## 1. Introduction

Oral cancer (OC) is the 15th most commonly diagnosed cancer worldwide and accounts for 2.1% of all cancers [1]. OC is a silent, invasive disease, which usually presents as a persistent, painless ulcer on the side of the tongue, or an intra-oral, red lesion without any disturbing symptoms. These signs are usually neglected by the patient and are sometimes unnoticed by dentists. The prognosis of OC depends on age, the general health of the patient, the type and location of the lesion, and the response to treatment [2]. Although there is limited evidence about the effectiveness of population-based visual screening on OC mortality, a reduction in mortality among patients at high-risk for OC has been reported [3]. Despite this limited evidence, it is important that dentists be on the lookout for any suspicious lesions while examining patients.

The incidence rate of OC varies by country due to differences in the distribution of etiological factors. In 2015, in Qatar, head and neck cancers accounted for 4% of all malignant cancers; about 60% of these cases were diagnosed in late stages (Qatar National Cancer Registry 2015, personal communication, November 2017). In addition, there has been an annual increase in the incidence of OC in the country, which has been linked to rapid population increases in the last decade. The population of Qatar consists of a high proportion of expatriates, and there is an imbalance in sex distribution, with a 3:1 male-to-female ratio [4].

The World Health Organization (WHO) Global Oral Health programs include two approaches to prevent OC: Reduction of exposure to risk factors and early detection through screening [5]; dental professionals play a crucial role in the latter. Several studies have evaluated healthcare professionals’ knowledge of OC and their clinical practice regarding OC examination [6,7,8,9,10]. Dentists in Qatar are of different nationalities and from different educational and training backgrounds. The aim of this study was to assess the existing knowledge on OC, and dentists’ attitude and clinical practice toward OC examination. In addition, we analyzed associations between oral cancer knowledge and the basic characteristics of dentists practicing in the governmental health sector in the State of Qatar.

## 2. Material and Methods

We invited all 271 dentists practicing in Qatar’s 23 Primary Health Care Centers (PHCC), which provide mainly general dental services, and the Hamad Medical Corporation (HMC), which provides a wide range of specialized treatment, to participate in this cross-sectional study. These practice settings were then divided into 5 clusters: PHCC-Northern region, PHCC-Central region, PHCC-Western region, HMC-Hamad Dental Centre, and HMC-Al Wakra Hospital dental clinics. Dentists were invited via their institutional e-mails; invitations included information about the study and encouraged dentists to participate. Non-responders were sent multiple additional invitations in an effort to boost recruitment. Those who chose to participate were asked to complete a structured, self-administered questionnaire (Appendix A), which was written in English. Of the 271 invited dentists, 177 completed and returned the questionnaire and were included in the analysis (response rate 65.31%). Completed questionnaires were sealed in envelopes coded with serial numbers to ensure privacy. Consent forms were obtained and included language on the assurance of confidentiality. Ethical approval was obtained from the Institution Review Board of Qatar University, on 11 March 2017 (No: QU-IRB 818-E/17) and from the Institution Review Board of the Primary Health Care Corporation, on 21 January 2018 (No: PHCC/IEC/17/12/043). HMC approval was obtained from Hamad Dental Centre. All participants signed an informed consent form, and confidentiality of all information was ensured by the secure storage of data in a password protected computer.

The questionnaire (Appendix A) consisted of four sections: (1) Personal characteristics, (2) knowledge of the clinical presentation of and risk factors for OC, (3) attitude toward OC examination, and (4) clinical practice regarding OC. There were six items in the personal characteristics’ section of the questionnaire. There were 14 items on the knowledge of the clinical presentation of OC and 16 items on the knowledge of risk factors for OC. Participants received 1 point for each correct response and were categorized by the number of correct responses (knowledge of the clinical presentation of OC: 0–9 low, 10–11 medium, 12–14 high; knowledge of the risk factors for OC: 0–8 low, 9–10 medium, 11–16 high) according to a previously developed scale [7,8]. This categorization was then further separated into satisfactory (medium/high) and unsatisfactory (low).

The section on attitude toward OC examination consisted of 11 items meant to measure dentists’ opinion about their role in OC prevention. Responses were given on 5-point Likert scale (1 = strongly agree, 2 = agree, 3 = disagree, 4 = strongly disagree, 5 = don’t know). The section on clinical practice regarding OC consisted of three items on the appropriate procedure to examine the oral cavity, factors dentists probe when talking to patients about their medical history, and the type of educational materials dentists had available for their patients. Responses to items in the attitude toward OC examination and clinical practice regarding OC sections of the questionnaire were measured as proportions. All questionnaire items were previously tested for validity and reliability [6,8].

## 3. Statistical Analysis

**Figure 1:**
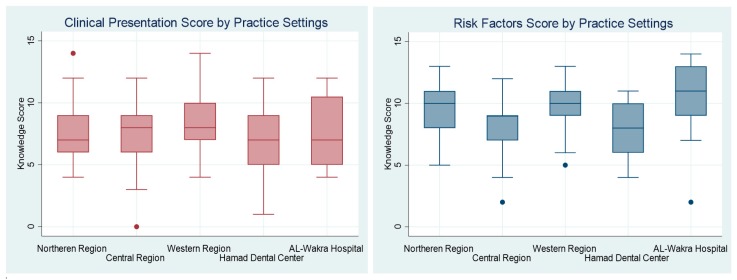
Score for knowledge of the clinical presentation of and risk factors for oral cancer by the five practice settings (*n* = 177).

Cluster-adjusted analysis was performed to account for the difference in the knowledge scores in the five different practice settings (Figure 1). The intra-class correlation coefficient (ICC) was used to adjust for the impact of a clustering effect on the knowledge of OC. We assessed the impact of personal characteristics (sex, years of practical experience, scope of practice, and last continuous professional development (CPD) course on OC attended) on the knowledge of OC using cluster-adjusted Chi^2^ and cluster-adjusted logistic regression. The cluster-adjusted Chi^2^ [11,12] is used for comparing dichotomous scores when the data include intra-cluster correlation. It calculates two adjusted chi-squared values, one based on the effect of clustering pooled across all participants’ settings, and one based on the effects of clustering by each setting. This method was applied to evaluate associations between the scores for knowledge of the clinical presentation and risk factors for OC and a number of covariates. Finally, univariate logistic regression, adjusted for the clustering effect, was used to identify potential predictors for satisfactory knowledge versus unsatisfactory knowledge of OC. All statistical analysis was performed using the statistical package, STATA 15 [13].

## 4. Results

### 4.1. Personal Characteristics

Of the 177 participating dentists, almost half were females. A total of 91 dentists (52.3%) had more than 15 years of practical experience, and just over half (102, 58.6%) were general practice dentists. Among the respondents, 62 dentists (35%) had attended a CPD course on OC within the past 2 years (Table 1).

### 4.2. Knowledge of the Clinical Presentation of and Risk Factors for Oral Cancer

The average score for knowledge of the clinical presentation of OC was 7.59 (SD = 2.40, range: 0–14). The majority of dentists could correctly identify proper tongue examination steps (147, 83.1%) for OC. A high proportion of dentists (149, 84.2%) correctly replied that squamous cell carcinoma is the most common form of OC, and 138 (77.9%) and 96 (54.2%) dentists correctly identified the tongue and the floor of the mouth as the first and second most common sites of OC, respectively. However, only 42.9% correctly identified both sites. Only 38 (21.5%) dentists replied correctly that early-stage OC is asymptomatic, and 56 (31.6%) dentists correctly replied that the majority of OC is diagnosed in people aged 60 years or older. One hundred and thirty-four (75.7%) dentists replied correctly that the lymph nodes are an important site of OC metastasis. OC is diagnosed mainly in advanced stages, but only 69 (39%) dentists were familiar with this. Leukoplakia and erythroplakia are conditions associated with OC, and 95 (53.7%) dentists correctly identified these conditions, but only 44 (24.9%) were able to define erythroplakia as a more serious premalignant condition than leukoplakia (Table 2).

**Figure 2:**
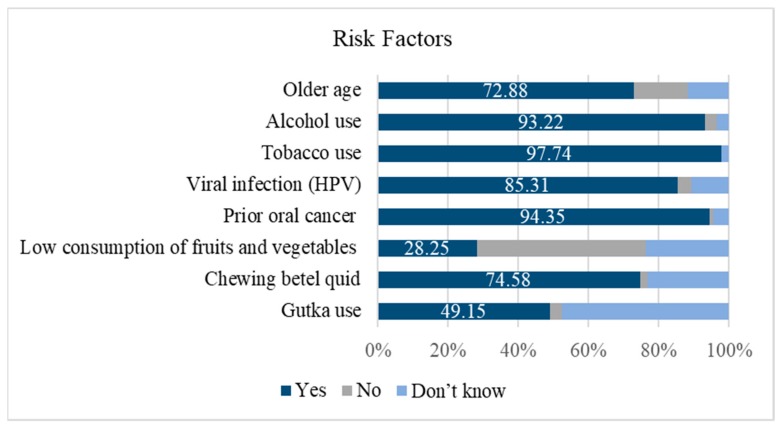
Percentage of dentists that correctly identified the listed risk factors for oral cancer (*n* = 177).

The average score for knowledge of the risk factors for OC was 8.96 (SD = 2.31, range: 2–14). A high percentage of dentists correctly identified older age (72.9%), alcohol use, (93.2%), tobacco use (97.7%), viral infection (85.3%), and prior OC (94.4%) as risk factors for OC. Fifty (28.3%) dentists correctly identified low consumption of fruits and vegetables as a risk factor for OC. Although 132 dentists (74.6%) correctly identified chewing betel quid as a risk factor for OC, 41 (23.2%) did not know of its effect. This was also seen with Gutka use, with 87 dentists (49.2%) correctly identifying it as a risk factor and 84 (47.5%) reporting that they did not know of its effect (Figure 2).

**Figure 3:**
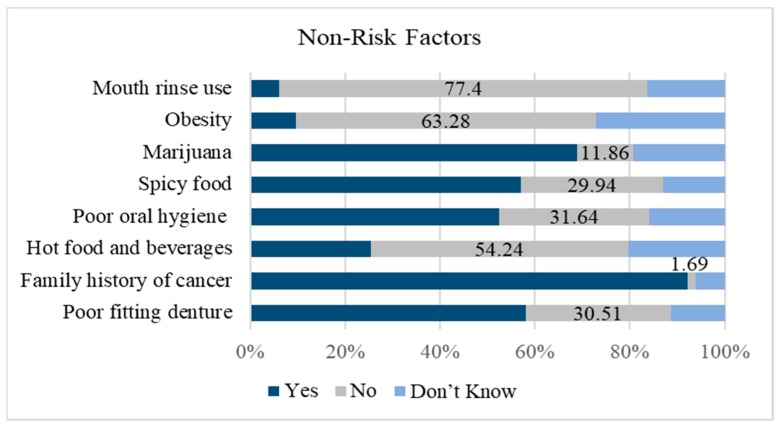
Percentage of dentists that correctly identified the listed non-risk factors for oral cancer (*n* = 177).

The majority of dentists (137, 77.4%) correctly identified mouth rinse use as a non-risk factor. However, only a low percentage of dentists correctly replied that spicy food (29.9%) and poor oral hygiene (31.6%) are non-risk factors for OC, and only 54 dentists (30.5%) correctly identified a poor fitting denture as a non-risk factor for OC (Figure 3).

To further explore patterns in the distribution of knowledge among dentists, knowledge of the clinical presentation of and risk factors for OC were cross-classified. Only 15.8% of dentists had satisfactory (medium/high) knowledge for both of these aspects; 30.5% had unsatisfactory (low) knowledge of both aspects. One hundred and thirty-eight dentists (78%) had unsatisfactory knowledge of the clinical presentation of OC.

### 4.3. Attitude Toward Oral Cancer Examination

**Figure 4:**
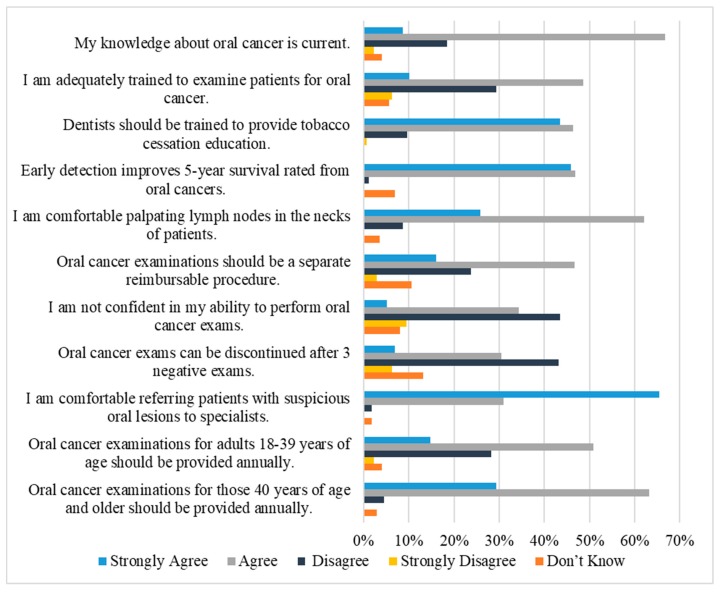
Dentists’ attitudes toward oral cancer examination (*n* = 177).

Among the respondents, 116 dentists (66.7%) agreed that their knowledge about OC was current. Also, 86 dentists (48.6%) agreed that they were adequately trained to examine patients for OC. The vast majority of dentists (90%) agreed that they should be trained to provide tobacco cessation education. Replies regarding dentists’ confidence in performing OC examination showed that 69 dentists (39.4%) either agreed or strongly agreed that they were not confident in their training. Moreover, 65.5% strongly agreed that they were comfortable referring patients with suspicious oral lesions to specialists (Figure 4). When respondents were asked about detecting the early signs and symptoms of OC, 80.39% of general practice dentists and 70.83% of specialist dentists believed that general practice dentists have the primary role. 

### 4.4. Clinical Practice Regarding Oral Cancer

When evaluating clinical practice, a majority of dentists were familiar with the proper physical oral examination steps (182 dentists, 72.3%). Moreover, 96% of dentists said that when taking a patient’s medical history, they ask about current tobacco use, 89.2% said they ask about previous tobacco use, and 80% reported asking about the type and amount of tobacco used. To a lower extent, dentists reported asking patients about their current alcohol use (68%), past alcohol use (64.7%), and type and amount of alcohol used (46.8%). Dentists also reported asking about their patients’ history of cancer (86.8%) and family history of cancer (80.6%). 

A high proportion of dentists (77.6%) reported that they have no educational material on OC available for their patients. Thirty-two dentists (18.4%) reported that they had brochures or pamphlets on OC available for their patients. Only three dentists (1.7%) reported that they provide verbal education and instructions to their patients about OC. 

### 4.5. Assessments of Dentists’ Personal Characteristics and Knowledge of Oral Cancer

The ICC was measured to evaluate differences in scores for the knowledge of the clinical presentation of and risk factors for OC across the five practice setting. The ICC for knowledge of the clinical presentation of OC was 0.028, and for the knowledge of risk factors of OC it was 0.084. Thus 2.8% and 8.4% of the variance in these scores is due to differences between the practice settings.

The distribution of low, medium, and high knowledge of the clinical presentation of and risk factors for OC showed that dentists aged less than 39 years were more likely to have high knowledge of the clinical presentation of OC when compared with older participants (adjusted Chi^2^ = 3.96, degrees of freedom [df] = 4, *p* value = 0.411). Dentists with less than 10 years of experience tended to have high knowledge of the risk factors for OC when compared to dentists with more than 10 years of experience (Adjusted Chi^2^ = 11.38, df = 4, *p* value = 0.023) (Table 3).

Cluster-adjusted univariate logistic analysis showed that dentists with less than 10 years of experience were 1.64 times more likely to have satisfactory knowledge of the clinical presentation of OC when compared to dentists with more than 15 years of experience. Also, dentists with less than 10 years of experience were 1.73 times more likely to have satisfactory knowledge of the risk factors for OC when compared to dentists with more than 15 years of experience, after adjusting for a clustering effect (Table 4). This significant association was not observed after applying the adjusted Chi^2^ test in Table 3; this is explained by the small sample size and the amended categorization of the study outcomes. The odds for dentists in specialty practice to have satisfactory knowledge of the clinical presentation of OC was 1.92 times that of dentists in general practice. The opposite was observed for knowledge about the risk factors for OC, with dentists in specialty practice being 46% less likely to have satisfactory knowledge. Dentists who attended a CPD course on OC within the past 2 years were 2.23 times more likely to have satisfactory knowledge of the clinical presentation of OC. No significant association was identified between attending CPD courses and knowledge of the risk factors for OC (Table 4). 

## 5. Discussion

A patient’s visit to the dentist is an opportunity for a comprehensive oral examination and a chance for one-on-one oral health education for the patients. The number of OC patients in Qatar has been increasing as the population has increased [4]. Qatar has a unique population structure with diverse ethnic origins, and these ethnic differences have been linked to the adoption of different health behaviors [14]. Qatar has universal healthcare coverage through the established governmental healthcare system, as all residents in the country are covered by the HMC and PHCC. Healthcare providers, and dentists in particular, play an important role in patient awareness and early detection of OC. This requires knowledge about the clinical presentation of and risk factors for OC. 

In dental practice, extra- and intra-oral examination is an essential step in the examination of a new patient [15]. In our study, a high percentage of dentists, higher than percentages observed in other studies, correctly identified the importance of lymph nodes in metastatic cancer [6,8]. This was reflected in their attitude, as most dentists agreed that they were comfortable palpating patients’ necks for a lymph node examination. An intra-oral examination includes a comprehensive examination of the oral cavity and the tongue. In our study, a large percentage of dentists demonstrated knowledge about the specific steps of oral and tongue examinations. However, a low percentage of dentists were confident in performing OC examinations. Moreover, only 48.6% of dentists felt that they had been adequately trained for this. Therefore, it is important that they receive regular training to refresh their basic knowledge and improve their oral and tongue examination skills. This could increase their confidence, and lead to a more positive attitude toward comprehensive oral examinations for their patients.

A high percentage of our dentists correctly recognized squamous cell carcinoma as the most common form of OC, which is similar to the percentage observed among dentists in Yemen, Kuwait, and North Carolina [7,10,16]. However, some important gaps in knowledge about the clinical features of OC were identified. Less than one-quarter (21%) of dentists correctly replied that OC is asymptomatic in its early stages, which is lower than what has been reported in Iran (45%) and British Columbia and Nova Scotia (78%) [6,8]. Accordingly, a low proportion of dentists (39%) replied that OC is mainly diagnosed at advanced stages, which is lower than what was observed in Kuwait (75%) and Yemen (66%) [10,16]. Almost half of the dentists (53.7%) were familiar with erythroplakia and leukoplakia as important signs of a premalignant lesion, but among them only 44 dentists (24%) correctly identified erythroplakia as a more serious premalignant condition. Similarly, in Iran, 50% of dentists correctly identified these signs. On the other hand, a higher percentage of dentists in Turkey (64.1%) and Kuwait (93%) correctly identified erythroplakia and leukoplakia as premalignant signs [10,17]. These are important signs of premalignancy that dentists need to know. The wide range in the level of dentists’ knowledge by the country of practice might be related to different educational backgrounds, different training opportunities, and different shared educational environments, considering the differences in the study periods. The gaps we identified in clinical and diagnostic knowledge of OC need to be addressed through CPD courses. 

A high percentage of dentists correctly identified tobacco, alcohol, and prior OC as risks for OC, which is consistent with other studies [6,16,18]. This was reflected in their practice of talking to patients about their medical history, as a majority of dentists properly assessed patients’ present tobacco use, history of cancer, and, to a lower extent, patients’ present alcohol use. Knowledge of the effect of chewing betel quid was poor, as almost half of the dentists did not know that Gutka use is a risk factor for OC. More emphasis is needed to increase their knowledge about the risk of different types of tobacco use, including smokeless tobacco use, which is widespread in Asian cultures. A high proportion of dentists believe that they should be trained to provide tobacco cessation education. Their knowledge that squamous cell carcinoma is directly linked to tobacco use showed their willingness to be actively involved in smoking cessation intervention.

Dentists had some problems distinguishing between risk factors and non-risk factors for OC. Therefore, more emphasis on the risk factors and non-risk factors for OC is needed in CPD courses. Increasing dentists’ knowledge of these factors would help to raise population awareness and play an important role in OC prevention. 

In the present study, nearly one-third of dentists had low knowledge of both the clinical presentation of and risk factors for OC. Moreover, 78% had low knowledge of the clinical presentation of OC. This is surprisingly more than what was observed in Clovis et al. (35%), Patton et al. (59%), and in Maryland (35.5%) [7,8,18]. Although 66.7% of our study participants reported that their knowledge of OC was current, this was not reflected in their level of knowledge on the clinical presentation of OC, indicating the need for educational intervention in Qatar. 

Dentists who had attended a CPD course on OC within the past 2 years were two times more likely to have satisfactory knowledge of the clinical presentation of OC. Although this association did not reach statistical significance, it was consistent with previous studies, in which attending a CPD course on OC significantly translated into better OC examination practices, better knowledge of the risk factors for OC among patients, and a higher level of clinical knowledge [19,20]. Furthermore, we observed no association between attending a CPD course and the level of knowledge on risk factors. This suggests that OC risk factors are not well addressed in the delivered courses, and that the focus is instead directed toward clinical knowledge. This is consistent with the results of Yellowitz et al., who showed that attending a CPD course on OC is not significantly associated with knowledge of the risk factors for OC [20]. This demonstrates that more emphasis is needed on the techniques used to deliver CPD courses and the importance of blended techniques of learning and education. CPD is regulated by Qatar Council for Health Care Practitioners (QCHP), and is compulsory for all healthcare practitioners in Qatar. Practicing dentists are expected to do 40 h of CPD every two years as a requirement for a practicing licensing renewal. However, it does not restrain part of the 40 h of CPD on a specific topic. Regarding the impact of years of experience, dentists with less than 10 years of experience were more likely to have high knowledge of the clinical presentation of and the risk factors for OC, compared to dentists with more than 15 years of experience, which is consistent with other studies [7,18]. Undergraduate studies have a positive impact on the level of knowledge, but this impact diminishes with time, reiterating the importance of CPD courses to update information. Currently, oral cancer screening in Qatar is not a separate reimbursement procedure [21]. It is part of the regular oral examination of patients. Qatar achieved universal coverage through the established governmental healthcare system, as all residents are covered with governmental health insurance in HMC and PHCC, including the oral examination procedure. However, a separate OC screening reimbursed procedure would improve the screening rate [22].

The strength of this study is represented by its methods. We used a paper-based, self-administered questionnaire to make it easy for participants to complete. Although this method is more costly and time consuming, we believe that it overcomes the accessibility barriers of our target population. The other strength of this study is the acceptable response rate of 65.31%, which is explained by the strategy we used to maximize response, including initial invitation well in advance of the study, followed by multiple additional invitations to non-responders.

Non-response bias is one of the limitations of this study. Indeed, non-respondents to this survey might have a different level of knowledge than respondents. However, there was a high proportion of respondents from the PHCC, and we believe there is no difference between the respondents and non-respondents from the PHCC. In the HMC, the response among dentists was poor, and thus respondents might be different than non-respondents. Another limitation is that dentists practicing in the private sector were not included; therefore, our results cannot be generalized to the private sector.

## 6. Conclusions

Assessing dentists’ knowledge is one way to measure their performance. This study identified gaps in knowledge among dentists practicing in the governmental health sector, which strongly suggested that dentists in Qatar could benefit from educational interventions (i.e., CPD courses) about the main clinical features of OC and the evidence-based risk factors. Our results showed that dentists with less than 10 years of practical experience had high knowledge of OC, thus one solution could be for dentists who have recently completed their undergraduate studies to get involved and share their knowledge by providing lectures or educational courses to colleagues in their practice settings. Dental offices are also a unique setting in which cost-effective tobacco cessation intervention could be provided. However, we recommend further research on the applicability of this type of intervention and the readiness of dentists to provide it. The results of this study can be used as baseline data for future CPD courses on OC for dentists and could help evaluate the effectiveness of such courses in raising dentists’ knowledge and awareness. Moreover, the study results can be used to plan future CPD courses aimed at improving the delivery of care in the health system.

## Figures and Tables

**Table 1:** Personal characteristics of the participants.

Characteristics (*n* = 177)	Frequency (*n*)	(%)
**Practice Setting**		
HMC	45	25.4%
PHCC	132	74.6%
**Sex**		
Male	92	51.9%
Female	85	48.1%
**Age (Years)**		
23–29	7	4.00%
30–39	83	47.4%
40–49	57	32.6%
50–59	22	12.6%
>60	6	3.40%
**Years of Practical Experience**		
<5 years	4	2.30%
5–10 years	31	17.8%
11–15 years	48	27.6%
>15 years	91	52.3%
**Scope of Practice**		
General Practice	102	58.60%
Specialty Practice	72	41.40%
**Last CPD Course on Oral Cancer Attended**		
<2 years	62	35.0%
2–5 years ago	39	22.0%
>5 years ago	50	28.3%
Never	26	14.7%
**Practice Setting**		
Northern Region	41	23.2%
Central region	45	25.4%
Western region	46	26%
Hamad Dental Centre	33	18.6%
AL-Wakra Hospital	12	6.8%

CPD: continuous professional development.

**Table 2:** Proportion of dentists that correctly identified the clinical presentation of oral cancer.

Clinical Presentation Questions (*n* = 177)	Number	(%)
Correctly identify tongue examination.	147	83.1%
Squamous cell carcinoma is the most common form of oral cancer.	149	84.2%
Tongue is the first most common site of oral cancer.	138	77.9%
Floor of the mouth is the second most common sites of oral cancer.	96	54.2%
Familial clustering is Least likely associated with oral cancer.	58	32.8%
Oral cancer early sign is asymptomatic.	38	21.5%
Majority of oral cancer cases are diagnosed in people 60 years or older.	56	31.6%
Lymph nodes Hard, painless, mobile or fixed.	134	75.7%
Ventral-lateral border of the tongue is site most likely develop oral cancer.	82	46.3%
Oral cancer most often diagnosed in advanced stage.	69	39.0%
Lip cancers are related to sun exposure.	112	63.3%
Early oral cancer lesions appear small, painless, red area.	118	66.7%
Erythroplakia and Leukoplakia are associated with oral cancer.	95	53.7%
Erythroplakia is a more serious premalignant condition than leukoplakia.	44	24.9%

**Table 3:** Association between dentists’ personal characteristics and knowledge of the clinical presentation of and risk factors for oral cancer (*n* = 177).

Knowledge of Clinical Presentation of Oral Cancer					Knowledge of the Risk Factors for Oral Cancer			
	Low Score (*n*)	Medium Score (*n*)	High Score (*n*)	*p* Value	Low Score (*n*)	Medium Score (*n*)	High Score (*n*)	*p* Value
**Sex**								
Male	73	12	7		32	35	25	
Female	65	16	4	0.671 ^†^	33	29	23	0.908 ^†^
**Age (years)**								
≤39	71	11	9		32	33	26	
40–49	45	11	0		24	19	13	
≥50	20	6	2	0.411 ^†^	7	12	9	0.603 ^†^
**Practical Experience (years)**								
≤10	25	7	4		9	11	16	
11–15	39	6	3		21	13	14	
>15	71	15	4	0.579 ^†^	33	40	17	0.023 ^†^
**Scope of Practice**								
General Practice	84	14	4		31	46	25	
Specialty Practice	51	14	7	0.825 ^†^	32	18	22	0.698 ^†^
**Last CPD Course on Oral Cancer Attended**								
<2 years ago	48	10	4		20	26	16	
2–5 years ago	26	10	3		14	12	13	
>5 years ago	41	5	4		23	12	15	
Never	23	3	0	0.912 ^†^	8	14	4	0.736 ^†^

CPD: continuous professional development.; ^†^ Cluster-adjusted Chi^2^ test.

**Table 4:** Univariate logistic analysis of the likelihood of having a satisfactory knowledge of oral cancer (*n* = 177).

Knowledge of the Clinical Presentation of Oral Cancer *				Knowledge of the Risk Factors for Oral Cancer *		
Characteristics	OR ^‡^	95% CI	*p* Value	OR ^‡^	95% CI	*p* Value
**Sex**						
Male	1.00	-		1.00	-	
Female	1.18	0.73–1.89	0.485	0.84	0.57–1.23	0.375
**Practical Experience (years)**						
>15 years	1.00	-		1.00	-	
11–15 years	0.86	0.19–3.78		0.744	0.41–1.33	
≤10 years	1.64	1.32–2.04	0.017	1.73	0.72–4.13	0.355
**Scope of Practice**						
General	1.00	-		1.00	-	
Specialty	1.92	0.91–4.03	0.084	0.54	0.22–1.33	0.184
**Last CPD Course on Oral Cancer Attended**						
Never	1.00	-		1.00	-	
>5 years ago	1.68	0.50–5.57		0.52	0.24–1.11	
2–5 years ago	3.83	0.82–17.8		0.79	0.30–2.06	
<2 years	2.23	0.40–12.3	0.321	0.93	0.57–1.51	0.329

* Binary outcome (satisfactory vs. unsatisfactory knowledge). ^‡^ Adjusted odds of satisfactory knowledge, accounting for clustering. OR: odds ratio, CI: confidence interval, CPD: continuous professional development.

**Figure A1:**
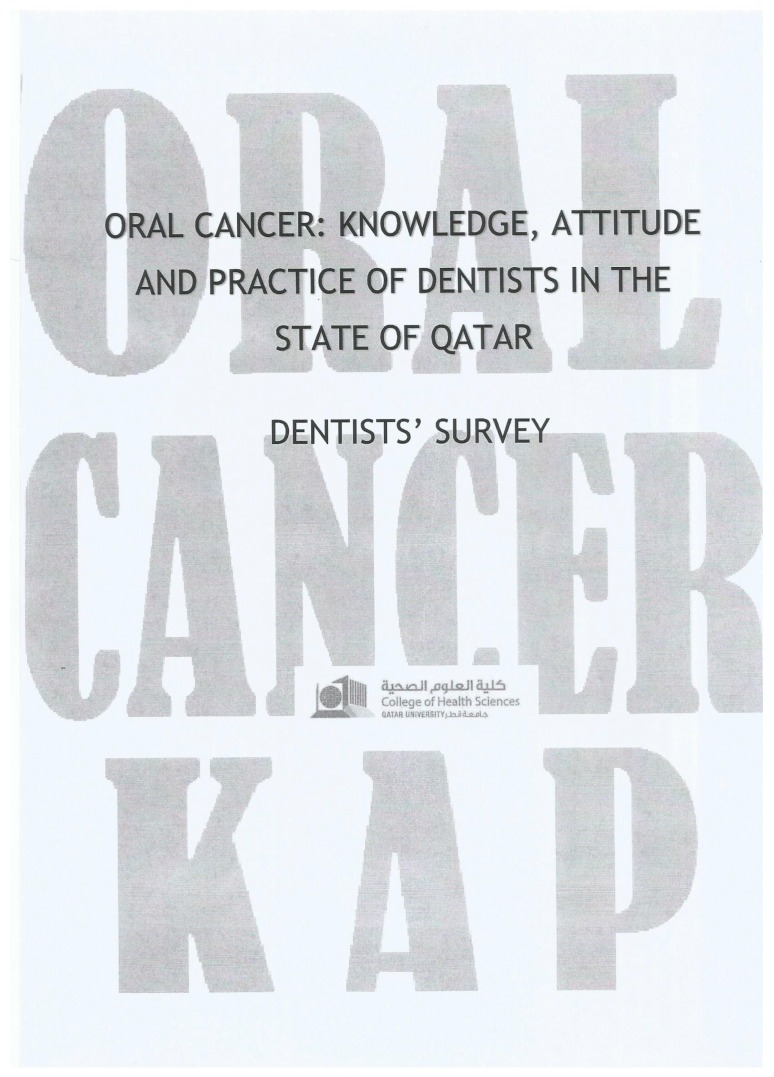

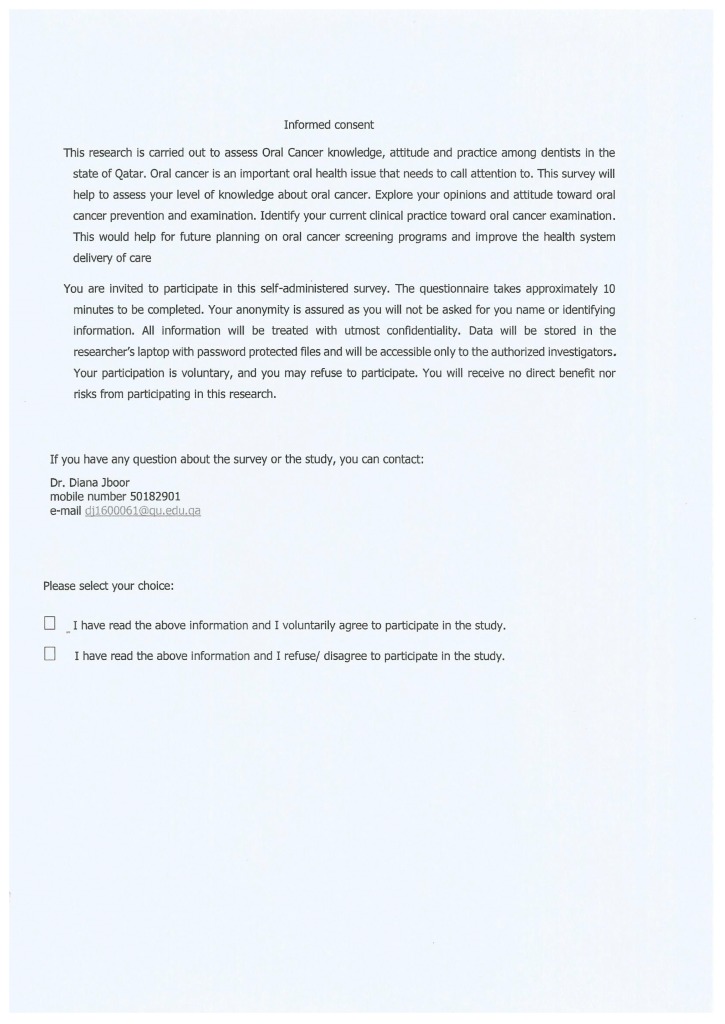

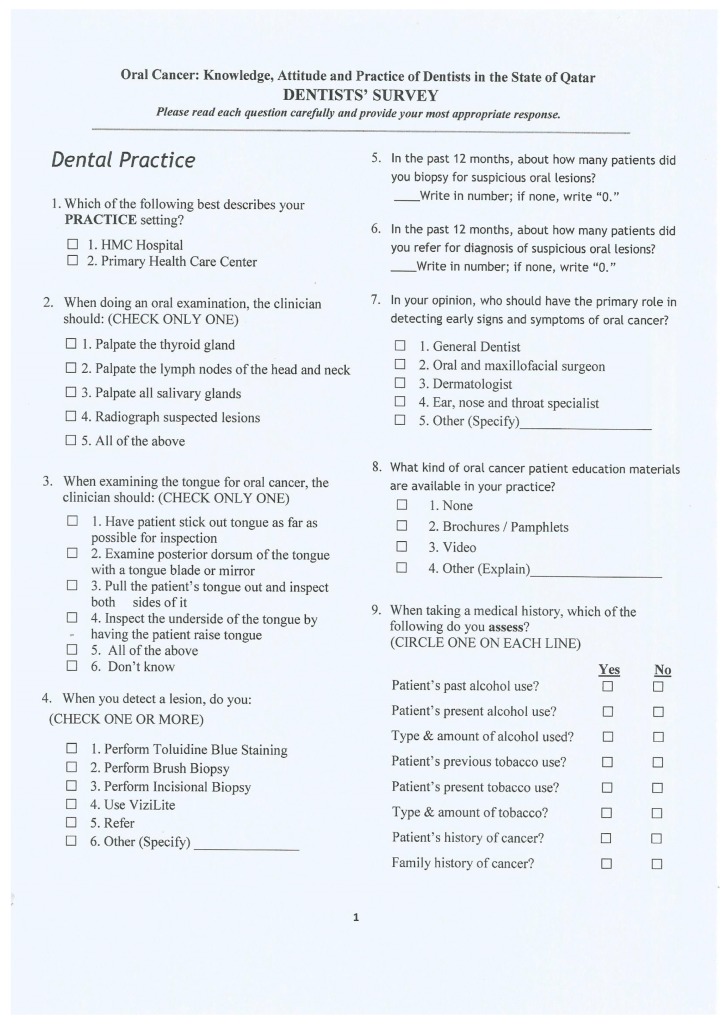

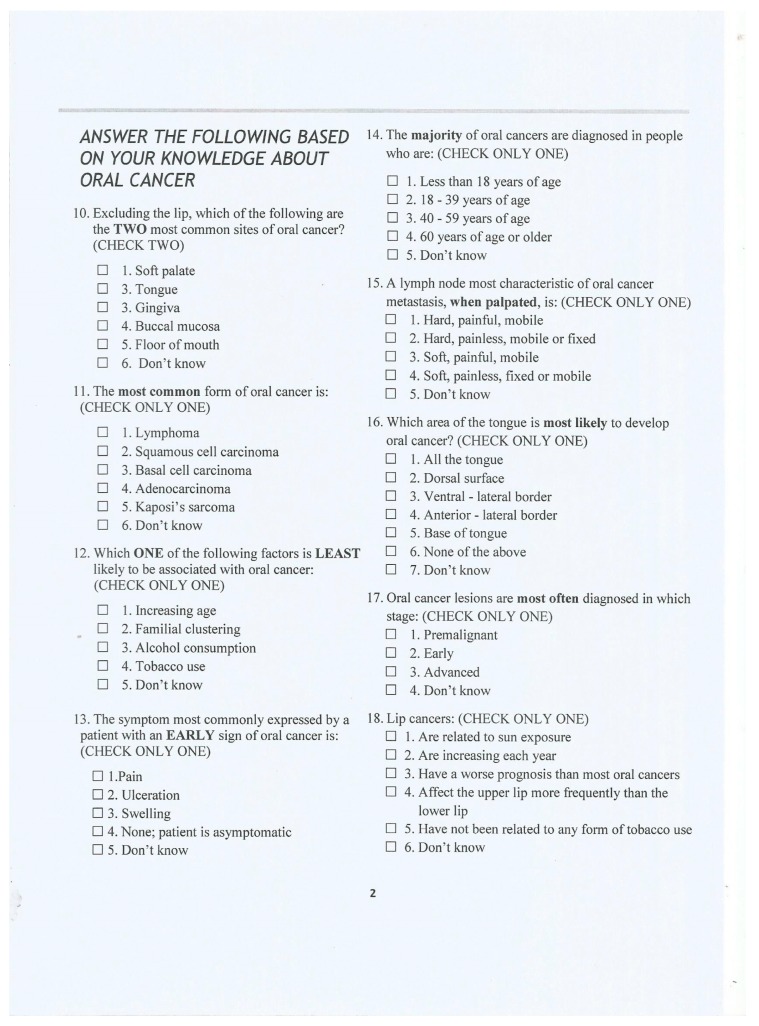

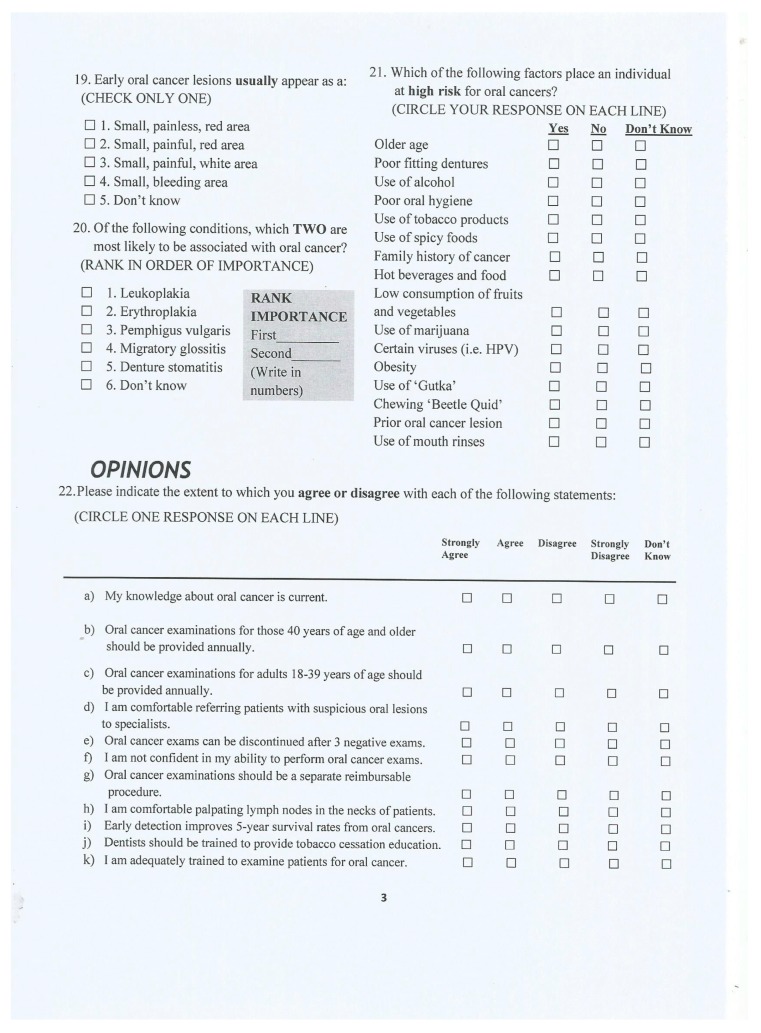

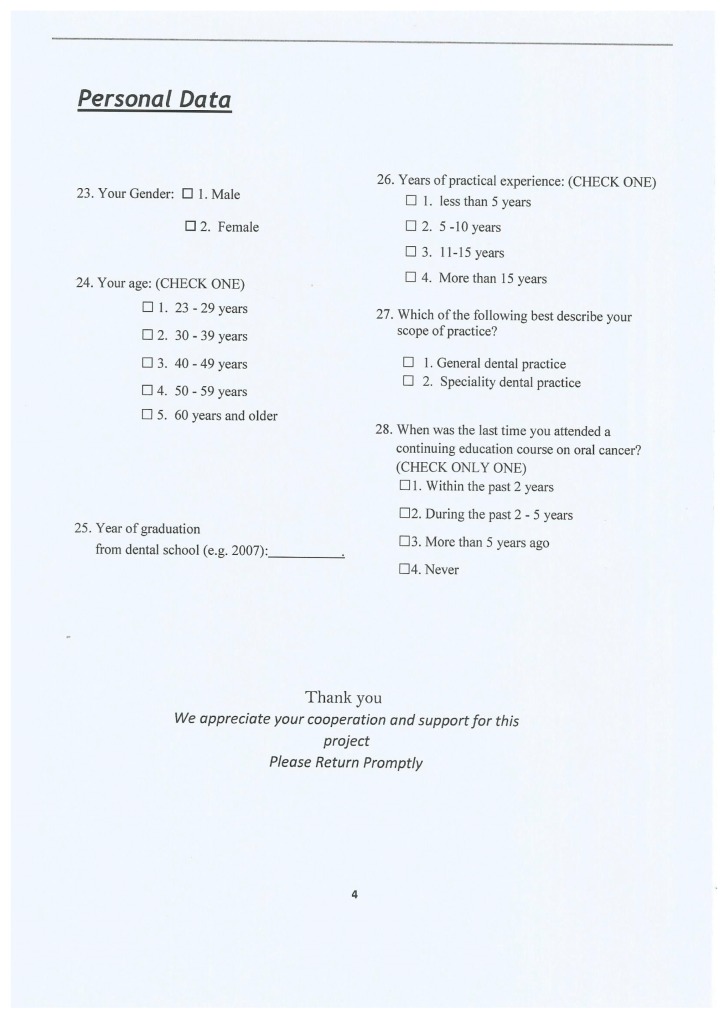
The Study Questionnaire.
